# Comparison of female rat reproductive effects of pubertal *versus* adult exposure to known endocrine disruptors

**DOI:** 10.3389/fendo.2023.1126485

**Published:** 2023-10-03

**Authors:** Julie Boberg, Tianyi Li, Sofie Christiansen, Monica K. Draskau, Pauliina Damdimopoulou, Terje Svingen, Hanna K. L. Johansson

**Affiliations:** ^1^ National Food Institute, Technical University of Denmark, Kgs. Lyngby, Denmark; ^2^ Division of Obstetrics and Gynecology, Department of Clinical Science, Intervention and Technology, Karolinska Institutet, Stockholm, Sweden; ^3^ Department of Gynecology and Reproductive Medicine, Karolinska University Hospital, Stockholm, Sweden

**Keywords:** endocrine disruption, risk assessment, reproductive toxicity, female reproduction, mode of action, alternative test methods

## Abstract

A prevailing challenge when testing chemicals for their potential to cause female reproductive toxicity is the lack of appropriate toxicological test methods. We hypothesized that starting a 28-day *in vivo* toxicity study already at weaning, instead of in adulthood, would increase the sensitivity to detect endocrine disruptors due to the possibility of including assessment of pubertal onset. We compared the sensitivity of two rat studies using pubertal or adult exposure. We exposed the rats to two well-known human endocrine disruptors, the estrogen diethylstilbestrol (DES; 0.003, 0.012, 0.048 mg/kg bw/day) and the steroid synthesis inhibitor ketoconazole (KTZ; 3, 12, 48 mg/kg bw/day). Specifically, we addressed the impact on established endocrine-sensitive endpoints including day of vaginal opening (VO), estrous cyclicity, weights of reproductive organs and ovarian histology. After 28 days of exposure, starting either at weaning or at 9 weeks of age, DES exposure altered estrous cyclicity, reduced ovary weight as well as number of antral follicles and corpora lutea. By starting exposure at weaning, we could detect advanced day of VO in DES-exposed animals despite a lower body weight. Some endpoints were affected mainly with adult exposure, as DES increased liver weights in adulthood only. For KTZ, no effects were seen on time of VO, but adrenal and liver weights were increased in both exposure scenarios, and adult KTZ exposure also stimulated ovarian follicle growth. At first glance, this would indicate that a pubertal exposure scenario would be preferrable as timing of VO may serve as sensitive indicator of endocrine disruption by estrogenic mode of action. However, a higher sensitivity for other endocrine targets may be seen starting exposure in adulthood. Overall, starting a 28-day study at weaning with inclusion of VO assessment would mainly be recommended for substances showing estrogenic potential e.g., *in vitro*, whereas for other substances an adult exposure scenario may be recommended.

## 1 Introduction

Exposure to chemicals from the environment may have negative effects on female reproductive development and, ultimately, women’s fertility. To protect against such detrimental effects, toxicological testing should ensure that chemicals on the market are safe for humans. This requires that the tests we use can detect potentially harmful chemical properties. However, recent publications have pointed out the concern that current test methods may not be sensitive enough to detect female reproductive toxicants (1, 2). This problem points to a need to clarify the optimal timing of exposure and sample collection, as well as an update of guidelines to include more sensitive endpoints.

Current chemical test guidelines (TGs) propose to screen for toxicity *in vivo* by a 28-day exposure study (3), or a screening study with reproductive performance combined with the 28-day study (4) using a small number of male and female rats. When validating the 28-day study (5) it was found not sufficient in identifying all endocrine disruptors acting by any of the four classical EATS modalities (estrogen, androgen, thyroid, and steroidogenesis). The assay only detected chemicals that were moderate or strong endocrine disruptors for (anti-) estrogenicity or (anti-) androgenicity (e.g., nonylphenol and DDE or ethinyl estradiol and flutamide, respectively). Thus, the 28-day study is not a sensitive screening assay for endocrine activity (3, 6). Specifically, the quality of data related to histological changes in cycling females was considered to depend significantly on the experience of the test laboratory, thus advice on how to conduct the histopathological assessments is provided in the guidance documents (GD) 43 and 106 (7, 8).

We hypothesized that the time of exposure could affect the sensitivity of the 28-day study. By initiating exposure at the time of weaning, sensitivity towards detecting effects on apical endpoints would increase as this early start would enable the examination of the day of vaginal opening (VO), which is known to be sensitive to endocrine disruption. An option to examine age at onset of estrous cycling was also explored. Before recommending such an amendment, however, it must be ascertained whether this early start will reduce the ability to detect other relevant effects. With this in mind, we aimed to compare the sensitivity of established endocrine sensitive endpoints in 28-day rat studies initiated either before or after puberty. We used the model compounds diethylstilbestrol (DES), a known potent estrogen and endocrine disruptor in humans (9, 10), and ketoconazole (KTZ), a fungicide that can perturb steroid hormone synthesis in humans and rodents by inhibiting various cytochrome P450 (CYP) enzymes of the steroidogenesis pathway, interfering with both androgen and estrogen synthesis (11–15). Using two compounds that we know have endocrine disrupting effects in humans enables comparison of the two exposure windows, as well as endpoint sensitivity using an established OECD test guideline (3). Perinatal exposure to these endocrine disruptors has been shown to affect reproductive development (1, 2, 16, 17) and they are expected to display different effect patterns depending on age at exposure start.

## 2 Materials and methods

### 2.1 Chemicals

Diethylstilbestrol (DES, CAS no. 56-53-1, purity ≥ 99%, cat.no. D4628) was purchased from Sigma Aldrich (St. Louis, MO, USA). Ketoconazole (KTZ, CAS no. 65277-42-1, purity 98%) was purchased from BOC Sciences Inc., USA. Corn oil was purchased from Sigma/Aldrich (cat. No. C8267-2.5L) and used as control and vehicle. Solutions were stored dark in glass bottles at room temperature and continuously stirred during the exposure period.

### 2.2 Animals and exposure

**Figure 1:**
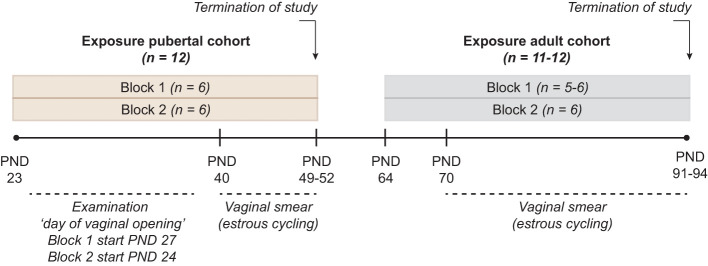
Study Design Two cohorts, exposed during pubertal (PND 23 to PND 49-52) or adult (PND 64 to PND 91-94) life stages were included. Each cohort was divided into two blocks where control group and all exposure groups were represented with n = 6/exposure group, giving a total n =12/exposure group in each of the two cohorts. Exposure to DES at doses of 0.003, 0.012, 0.048 mg/kg body weight (bw)/day, or KTZ at 3, 12, 48 mg/kg bw/day was conducted daily via oral gavage throughout the exposure period. In the pubertal cohort, day of vaginal opening and body weight at vaginal opening was registered. In both the pubertal and adult cohort vaginal smearing was conducted to evaluate estrous cycling. At the termination of study (both pubertal and adult cohort) body weight as well as the weight of ovaries, uterus, adrenal and liver was registered, and ovaries fixed in formaldehyde for histological evaluation. In the adult cohort, block 1, one animal in KTZ-48 was acutely euthanized few days before planned termination.

The animal study was conducted as described in (18). In short, time-mated nulliparous Sprague-Dawley rats (CD IGS Rat, Crl : CD(SD), Charles River Laboratories, Sandhofer Weg 7, Sulzfeld, Germany) were supplied on gestation day (GD) 3 to the animal facilities at the National Food Institute, Technical University of Denmark (DTU Food), Kgs. Lyngby, Denmark. Female offspring were weaned on postnatal day (PND) 22 and divided into two cohorts exposed either during puberty (n = 12 per dose group) or during adulthood (n=11-12 per dose group). To practically enable a group size of twelve/cohort, each cohort was exposed in two blocks with one week between the blocks. Control group and all exposure groups were represented in each block (n = 6 per dose group/block) (**Figure 1**). Animals were exposed by oral gavage from postnatal day (PND) 23 to PND 49- 52, or from PND 64 to PND 91-94. The exact day of study termination differed in a four-day interval to enable dissection at the di-estrous stage. Both cohorts were exposed to vehicle only (control groups), DES at doses of 0.003, 0.012, 0.048 mg/kg body weight (bw)/day, or KTZ at 3, 12, 48 mg/kg bw/day. DES and KTZ are known endocrine disruptors and were used as model compounds for proof of principle, hence the doses used were not chosen based on environmentally relevant exposure levels. The two lower doses reflect doses applied in a previous study using perinatal exposure (2). In the current study the high dose was added as it was expected to be better tolerated in the used exposure periods compared to exposure during gestation. The vehicle volume was 2 ml/kg, and the individual doses were based on body weight of the animal, assessed daily. One animal from the adult exposure study was euthanized during the study due to poor health condition. The animal experiments were approved by the Danish Animal Experiments Inspectorate (Council for Animal Experimentation, license number 2020-15-0201-00570) and monitored by DTU in-house Animal Welfare Committee.

### 2.3 *In vivo* examinations

In the pubertal cohort, vaginal opening (VO) was examined daily from PND 27 in the first block, but due to some animals already presenting with VO on PND 27, examination was initiated on PND 24 in the second block. The day of VO was registered together with the body weight. In the pubertal study vaginal smears were collected from PND 40 until study termination (PND 49-52), and in the adult cohort from PND 70 to study termination (PND 91-94) to evaluate estrous cyclicity as described in Johansson et al. (2). Regularity of estrous cycling was evaluated according to criteria based on Goldman et al. (19): regular cycling: 3–5 days cycle length with either 1-2 days of estrous or/and 2-3 days of di-estrous; irregular cycling < 3 days cycle length or ≥ 6 days cycle length, or/and with 3-4 days of estrous or/and 4-5 days of di-estrous; or prolonged cycling > 4 consecutive days of estrous or/and > 6 days of di-estrous. The person conducting scoring of estrous stage and evaluation of cycling regularity was blinded to animal exposure status. At the day of expected di-estrous, females were killed by decapitation (under CO2 anesthesia). Ovaries were excised and weighed and one ovary per female was fixed in 10% formaldehyde and processed for mounting in paraffin. Uterus, adrenals and liver were excised and weighed.

### 2.4 Histology and assessment of ovarian follicles and corpus luteum

Pubertal ovaries (n = 12/exposure group) were sectioned at 5 μm thickness every 100 μm and adult ovaries (n =11-12/exposure group) at 5 μm thickness every 200 μm. Paraffin was removed by heat treatment (60°C) for 30 min, then sections were washed in petroleum and hereafter rehydrated through a graded ethanol-water series. For histological evaluation, slides were stained with hematoxylin & eosin (H&E), dehydrated through a graded water-ethanol series, mounted using Eukitt. Histology images were captured using a Pannoramic Midi automatic digital slide scanner (3Dhistech Ltd). Digitalized H&E sections were annotated using Quantitative Pathology & Bioimage Analysis (QuPath version 0.2.3) software. Follicles were categorized into small antral follicles, growing antral follicles, or pre-ovulatory follicles using categories published by Gaytan et al. (20) (**Supplementary Table 1**) and described in our previous publication (18). Note that in the previous paper (18), the number of growing antral follicles and pre-ovulatory follicles were pooled into a category called antral follicles, and small antral follicles were not included in the evaluation (18), whereas in the present study, the categories used are as described above. The person conducting scoring was blinded with respect to dose group.

### 2.5 Statistical analysis

P-values ≤ 0.05 were considered statistically significant. Data were assessed for normal distribution by residual statistics. Data not normally distributed were log-transformed and assessed again to confirm normality. Body weights were analysed by ANOVA followed by Dunnett’s test using GraphPad Prism (GraphPad Software, San Diego, CA, US, version 8.0), and organ weights were analysed by ANCOVA using body weight as covariate (RStudio). Follicle and corpus luteum count was analysed by Kruskal-Wallis followed by Dunnett’s test using RStudio (4.0.5). Time-to-Event data endpoints (day of VO) were analysed by Kaplan-Meier methods, with general dose-related patterns identified by a trend test and paired mean differences between controls and dose group by the nonparametric Wilcoxon test under the control of p-value adjustments according to Dunnett (21). Data are reported as mean ± SEM in text. Analysis of estrous cycle data was conducted by comparing each exposure group to the control group using Fisher’s exact test in GraphPad Prism 8. The analysis was conducted in a stepwise manner where the first step pooled the irregular and prolonged categories (category named ‘altered cycling’) to make the analysis stronger. In the second step, the groups showing effect were further investigated, dividing the irregular and prolonged categories, analyzing them separately.

## 3 Results

### 3.1 Pubertal exposure

#### 3.1.1 Day of vaginal opening and estrous cyclicity

In the first exposure block examination of VO was initiated on PND 27, but as VO was already observed in some animals at this time point (in DES exposed groups), we initiated examination on PND 24 in the second exposure block, showing that some animals in DES-0.012 and DES-0.048 presented VO on PND 26. As significant effects were seen in both blocks compared to corresponding control, we pooled the results of the two blocks for the final statistical analysis. Compared to control, advanced day of VO and reduced body weight at VO was evident in all DES exposure groups (**Table 1**). The reduced body weight at VO can likely be attributed to the younger age of DES animals, which across DES groups were approximately 7.5 days younger than controls at VO. However, a potential effect of exposure cannot be neglected. No effect on day of VO or body weight at VO was observed in any of the KTZ groups (**Table 1**).

**Table 1:** Details on the day of vaginal opening (VO), body weight at vaginal opening and corresponding p-values in comparison to control group.

	Dose (mg/kg bw/day)	n	Day of VO (mean ± SEM)	p-value day of VO*	Body weight at VO mean ± SEM)	p-value body weight*
**Control**	0	12	35.5 ± 0.4		108.2 ± 3.4	
**DES**	0.003	12	28.3 ± 0.7	0.02	64.5 ± 3.2	< 0.0001
	0.01	12	27.4 ± 0.3	0.002	57.7 ± 1.3	< 0.0001
	0.048	12	27.2 ± 0.3	0.0001	60.1 ± 1.7	< 0.0001
**KTZ**	3	12	35.0 ± 0.7	0.6	106.4 ± 3.5	0.98
	12	12	36.2 ± 0.7	0.9	108.5 ± 4.1	1
	48	12	35.9 ± 0.4	0.6	111.1 ± 2.9	0.89

*p-values are from comparison with control group.

All DES exposed groups had significantly advanced day of VO and significantly lower body weight at VO (which is likely explained by them being much younger than controls at day of VO). No effects were seen after exposure to KTZ.

**Figure 2:**
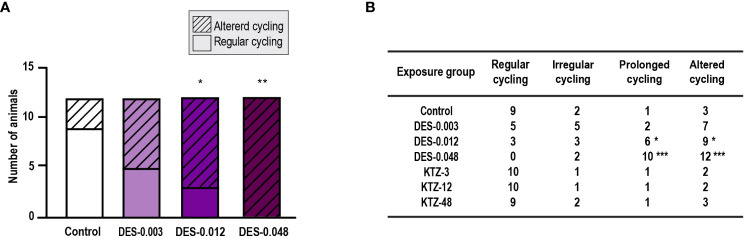
Estrous cycling, pubertal exposure. **(A)** Estrous cycling in the period from PND 40 to PND 50 was affected in female rats exposed to DES for 28 days starting at PND 23 (irregular and prolonged cycle categories pooled into one category; altered cycling), **(B)** The number of rats with prolonged (and altered) cycling increased with higher doses of DES, whereas cycling in KTZ rats appeared normal. Data analyzed by Fisher’s exact test (GraphPad Prism 8) (n=12/exposure group, * p < 0.05, ** p < 0.01, *** p < 0.001).

Estrous cyclicity was evaluated from PND 40, as animals younger than this were considered too small for vaginal smearing using cotton swabs. At this age, all except one control animal had started cycling (**Supplementary Figure 1**) and evaluation of potential differences in onset of estrous cycling was therefore not possible. However, regularity of estrous cycling was evaluated. Most of the animals in the DES-0.048 group showed a pattern of persistent estrous, which was also seen for several of the animals in the DES-0.012 group at the beginning of the assessment period (**Supplementary Figure 1**). By pooling irregular cycling and prolonged cycling into one category (altered cycling) we determined that the DES-0.012 and DES-0.048 groups were statistically significantly different from controls (p = 0.04, p = 0.0003 respectively), whereas DES-0.003 was not (p = 0.2) (**Figure 2A**). Further analysis, dividing the categories, showed that the effect was mainly driven by prolonged cycles (**Figure 2B**). No altered cycling was observed in KTZ exposed animals.

#### 3.1.2 Body and organ weights

**Figure 3:**
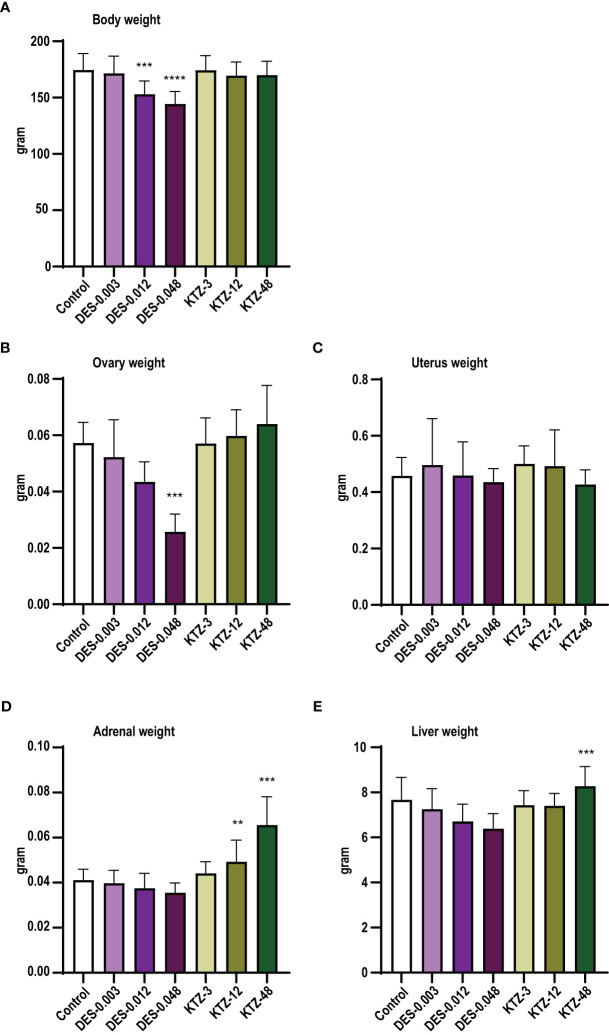
Body and organ weights, pubertal exposure. **(A)** body weight, **(B)** ovary weight, **(C)** uterus weight, **(D)** adrenal weight, **(E)** liver weight. DES reduced body and ovary weights, whereas KTZ increased adrenal and liver weights in female rats exposed for 28 days starting at PND 23. Body weights were analyzed by ANOVA followed by Dunnett’s test (GraphPad Prism), and organ weights were analyzed by ANCOVA using body weight as covariate (RStudio) (mean+SEM, n=12, ** p < 0.01, *** p < 0.001, **** p > 0.0001).

At termination, body weight was significantly lower in the DES-0.012 group (p = 0.001) and DES-0.048 group (p < 0.0001) compared to controls (**Figure 3A**), but no effect was seen after KTZ exposure. A significant reduction in ovary weight was seen in the DES-0.048 group (p = 4.5 · 10^-5^) (**Figure 3B**). Adrenal weights were significantly increased in the KTZ-12 group (p = 0.01) and KTZ-48 group (p = 8.2 · 10^-10^) (**Figure 3D**), whereas liver weights were increased in KTZ-48 group (p = 0.001) (**Figure 3E**). No effect was seen on uterus weight (**Figure 3C**).

#### 3.1.3 Follicle and corpus luteum count

**Figure 4:**
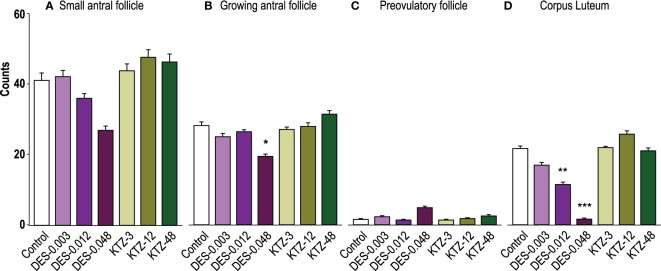
Follicle and corpus luteum count, pubertal exposure. **(A)** No statistically significant effect was seen on small antral follicles, **(B)** DES-0.048 significantly reduced the number of growing antral follicles, **(C)** No effect was seen on preovulatory follicles, and **(D)** DES-0.012 reduced the number of corpus luteum. Data were analyzed by Kruskal-Wallis test followed by Dunnett’s *post hoc* test in RStudio (4.0.5) (mean + SEM, n = 12, * p < 0.05, ** p < 0.01, *** p < 0.001).

**Figure 5:**
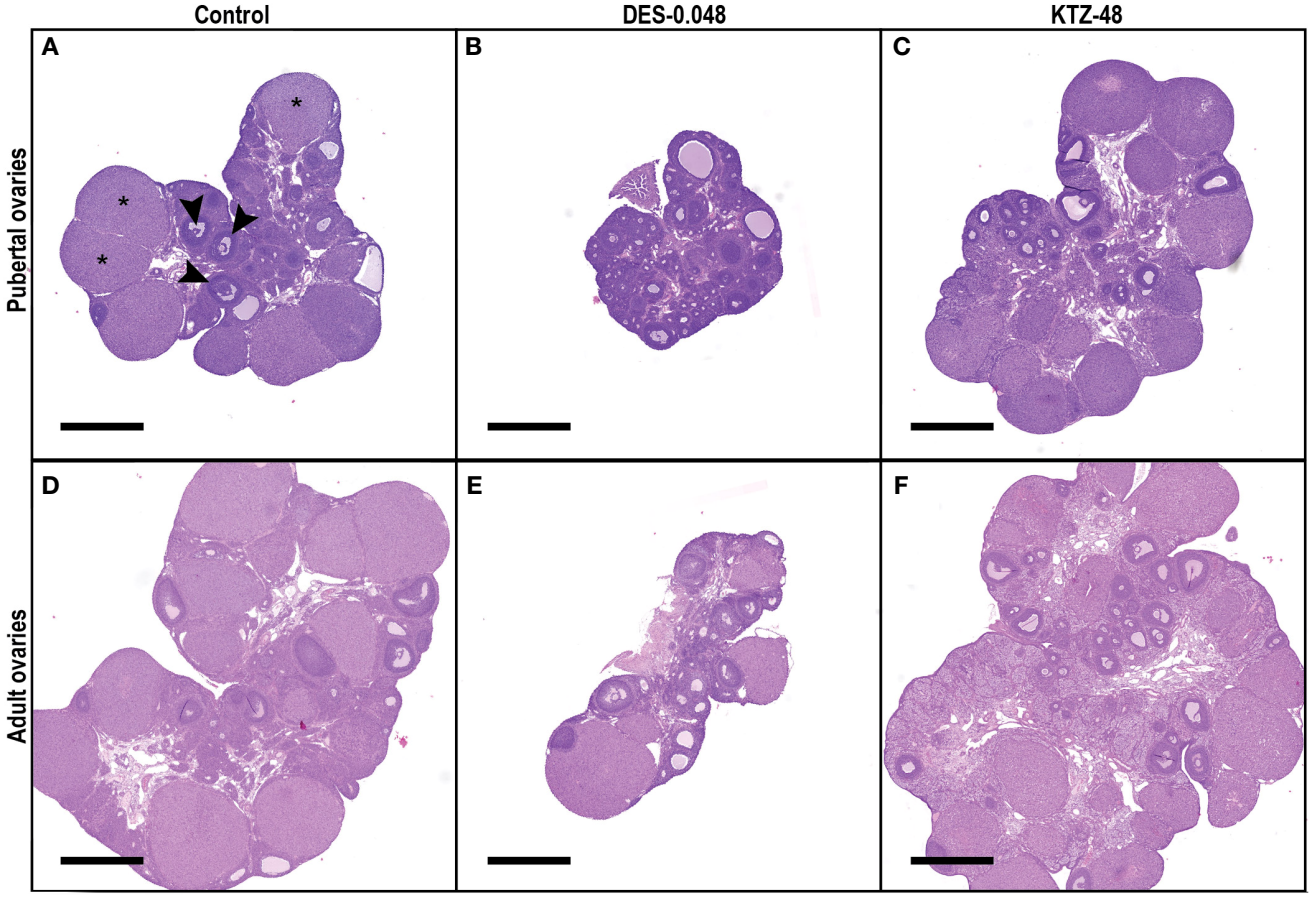
Histological sections of pubertal and adult ovaries. Representative sections from the middle of the ovary from pubertal **(A–C)** and adult **(D–F)** animals exposed to control **(A, D)**, DES-0.048 **(B, E)** or KTZ-48 **(C, F)**. In pubertal ovaries **(A–C)**, DES reduced ovary weight as well as the number of growing antral follicles and corpus luteum (CL) number, whereas no significant effects were seen after KTZ exposure. In adult ovaries **(D–F)**, DES reduced ovary weight, small antral follicles, growing antral follicles and CL numbers, whereas KTZ increased the number of growing antral follicles. In picture **(A)** three examples of CL are marked with stars (*) and three examples of growing antral follicles are marked with arrows (▸). The magnification is the same in all pictures and the scalebar is 1000 µm.

Ovaries from the DES-0.048 group had fewer growing antral follicles and corpora lutea (p = 0.038, and p < 0.0001, respectively, **Figures 4B, D**), which is consistent with reduced ovary weights (**Figure 3B**). Exposure to DES-0.012 led to significant decline of corpus luteum number (p = 0.027, **Figure 4D**), whereas the lower number of small antral follicles was not statistically significant (**Figure 4A**) and no effects were seen on preovulatory follicles (**Figure 4C**). No effects were seen for KTZ exposed animals. Histological sections from control, DES-0.048, and KTZ-48 are shown in **Figures 5A–C**.

### 3.2 Adult exposure

#### 3.2.1 Estrous cycling

**Figure 6:**
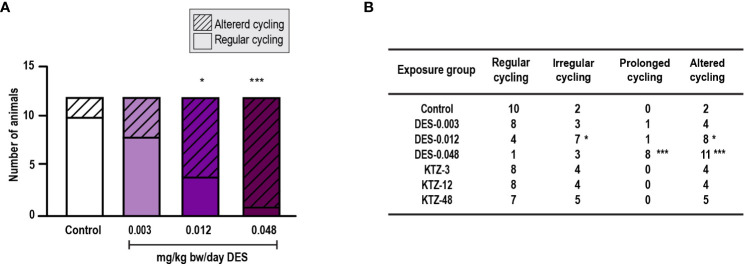
Estrous cycling, adult exposure. **(A)** DES exposure increased the number of females with altered cycles compared to controls in female rats exposed for 28 days starting PND 63. Irregular and prolonged cycle categories were pooled into one category (altered cycling) and analyzed by Fisher’s exact test (GraphPadPrism 8), **(B)** The number of rats with irregular or prolonged estrous cycles increased with higher doses of DES, whereas cycling in KTZ rats appeared normal (* p < 0.05, *** p < 0.001, n=12/exposure group).

Females exposed to DES during adulthood displayed clear changes in their estrous cycling. The DES-0.048 group mainly showed prolonged cycles where majority of females also displayed mucus in smears for more than 4 consecutive days, whereas animals in the DES-0.012 group showed varied cycling patterns (**Supplementary Figure 2**). Pooling irregular cycling and prolonged cycling into one category (altered cycling), showed increase in altered cycling among animals in both the DES-0.012 group (p = 0.04) and DES-0.048 group (p = 0.0006) compared to controls (**Figure 6A**), but no effect in the DES-0.003 group (p = 0.6). Further stepwise analysis showed that in the DES-0.048 group the effect was mainly driven by prolonged cycles and in the DES-0.012 group it was mainly due to irregular cycling patterns (**Figure 6B**).

#### 3.2.2 Body and organ weights

**Figure 7:**
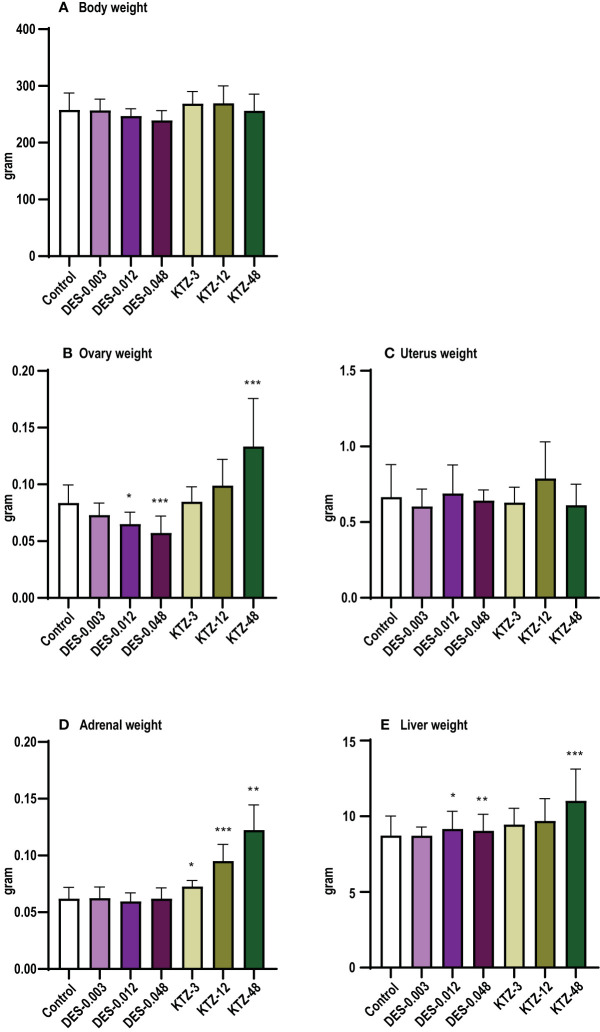
Body and organ weights, adult exposure. **(A)** body weight, **(B)** ovary weight, **(C)** uterus weight, **(D)** adrenal weight, **(E)** liver weight. DES reduced ovary weights and increased liver weights, whereas KTZ increased ovary, adrenal and liver weights in female rats exposed for 28 days starting PND 63. Body weights were analyzed by ANOVA followed by Dunnett’s test (GraphPad Prism), and organ weights were analyzed by ANCOVA using body weight as covariate (RStudio) (Mean + SEM; n=11-12, * p < 0.05, ** p < 0.01, *** p < 0.001).

As with pubertal exposure, ovary weights were significantly reduced in the DES-0.012 group (p = 0.02) and DES-0.048 group (p = 0.001) (**Figure 7B**). Slight effects were observed on body weights but were not statistically significant (**Figure 7A**). Liver weights were increased in the DES-0.012 group (p = 0.02) and DES-0.048 group (p = 0.008) (**Figure 6E**), an effect not seen with pubertal exposure. In contrast to what was seen with pubertal exposure, the KTZ-48 group displayed increased ovary weight (p = 3 · 10^-6^) compared to controls (**Figure 7B**). In addition, KTZ increased adrenal weight, and this was significant at all doses (KTZ-3: p = 0.04, KTZ-12: p = 1.5 · 10^-9^, KTZ-48: p = 8.8 · 10^-13^) (**Figure 7D**), whereas liver weights were increased at KTZ-48 only (1.2 · 10^-7^) (**Figure 7E**). No effects were seen on uterus weight (**Figure 7C**).

#### 3.2.3 Ovarian follicle and corpus luteum count

**Figure 8:**
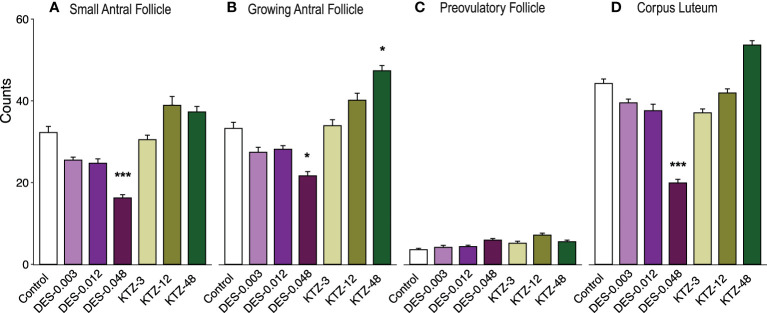
Follicle and corpus luteum count, adult exposure. **(A)** DES-0.048 significantly reduced the number of small antral follicles, **(B)** DES-0.048 significantly reduced the number of growing antral follicles and KTZ-48 significantly increased the number of growing antral follicles, **(C)** no effects were seen on preovulatory follicles, **(D)** DES-0.048 significantly reduced the number of corpus luteum. Data was analyzed by Kruskal-Wallis test followed by Dunnett’s *post hoc* test in RStudio (4.0.5) (Mean + SEM, n = 11-12. * p<0.05 *** p<0.001).

Consistent with the changes to ovary weights (**Figure 7B**), DES-0.048 reduced whilst KTZ-48 increased the number of growing antral follicles (p = 0.036, and p = 0.047, respectively, **Figure 8B**). Significantly lower numbers of small antral follicles and corpora lutea were also observed after DES-0.048 exposure (p = 0.0008, and p < 0.0001, respectively, **Figures 8A, D**). No effects were seen on preovulatory follicles (**Figures 8C**). Histological sections from control, DES-0.048, and KTZ-48 are shown in **Figures 5D–F**.

### 3.3 Comparison of effects and sensitivity

An overview comparing effects in the two exposure scenarios is presented in **Table 2**. Generally, the effects were similar in the two exposure scenarios, but it seems as if adult exposure was more sensitive in detecting effects on adrenal weight (for KTZ) and ovary weight (for DES), as these effects were seen at a lower dose than in the pubertal exposure scenario.

**Table 2:** Patterns of changes in organ weights and follicle/corpora lutea counts differed between the pubertal and the adult exposure scenarios, arrows indicate statistically significant increases or decreases.

	Pubertal			Adult		
**DES (mg/kg bw/day)**	**0.003**	**0.012**	**0.048**	**0.003**	**0.012**	**0.048**
**Vaginal opening**	advanced	advanced	advanced	NR	NR	NR
**Estrous cycling**		affected	affected		affected	affected
**Body weight**		↓	↓			
**Ovary weight**			↓		↓	↓
**Uterus weight**						
**Adrenal weight**						
**Liver weight**					↑	↑
**Small antral follicles**						↓
**Growing antral follicles**			↓			↓
**Preovulatory follicles**						
**Corpora lutea**		↓	↓			↓
**KTZ (mg/kg bw/day)**	**3**	**12**	**48**	**3**	**12**	**48**
**Vaginal opening**				NR	NR	NR
**Estrous cycling**						
**Body weight**						
**Ovary weight**						↑
**Uterus weight**						
**Adrenal weight**		↑	↑	↑	↑	↑
**Liver weight**			↑			↑
**Small antral follicles**						
**Growing antral follicles**						↑
**Preovulatory follicles**						
**Corpora lutea**						

NR, not relevant.

## 4 Discussion

One of the challenges in the field of endocrine disruption is that current test methods seem inadequate in detecting female reproductive toxicants, a problem that relates to optimal time of exposure, time of measurement as well as the endpoints that are investigated (1, 2). There is thus a need to optimize the testing strategy. In this study, we focused on the time of exposure by comparing endocrine sensitive endpoints in two 28-day studies initiated either before or after puberty. We hypothesized that starting a 28-day study before puberty would increase the overall sensitivity to detect chemicals with endocrine disrupting properties, and specifically chemicals with estrogenic properties, as it enables examination of the day of vaginal opening (VO). VO has previously been shown sensitive to different estrogenic chemicals such as BPA, mycotoxin (zearalenone) and phytoestrogens (22–25) as a well as DES (26, 27) after prepubertal exposure.

### 4.1 DES advanced vaginal opening

Starting the exposure before puberty gave us the opportunity to examine effects on the day of VO, which is currently not an endpoint included in the 28-day study guideline (3). DES clearly advanced the day of VO, which has previously been shown in rats exposed orally to DES from weaning (27), in rats subcutaneously exposed to DES from PND 12 (26), and recently in mice exposed prenatally (28). Other chemicals, such as BPA and the mycotoxin zearalenone, has also been shown to advance the day of VO (22–24). In these studies, central onset of puberty was also affected. Unfortunately, onset of estrous cycling (and indirectly first ovulation and central initiation of puberty) could not be evaluated in the present study as all DES animals had started cycling when smearing was initiated (PND 40). Also, vaginal tissue seems to be sensitive to the local chemical environment. Several studies have shown that local effects on VO, unrelated to central puberty onset, can be induced by treatment with estrogen (26), testosterone (26, 29, 30), dehydroepiandrosterone (31) and the cancer drug Dabrafenib (32). Furthermore, the vaginal tissue seems to contain aromatase activity, explaining induction of VO after androgen exposure (30). Due to this, we do not know if the advanced VO in the present study is a local effect or if central puberty was also affected. This shows that inclusion of VO in the 28-day study guideline as a ‘stand-alone parameter’ for sexual maturation, not including measurements related to central activation of puberty, is not advisable. However, as the vaginal tissue seems to be hormone (estrogen) sensitive, unrelated to central effects on puberty, VO alone could possibly serve as an effect endpoint indicative of endocrine disruption by estrogenic mode of action.

### 4.2 Comparison of effects in the two exposure scenarios - is pubertal 28-day exposure more sensitive than adult 28-day exposure*?*

Body weight changes at termination of the experiment were detected with pubertal, but not adult, exposure to DES. This could be explained by effects of DES on longitudinal growth and bone maturation which is under endocrine control. In females with precocious puberty, increased estrogen levels cause premature epiphysial fusion decreasing finale height (33). Also, growth hormone (GH) and IGFs are important for pubertal growth and interestingly, when estrogens are orally administered, they inhibit IGF-I production by the liver in a dose-dependent manner. As IGF-I normally mediates anabolic action of GH, the orally administered estrogen can attenuate the stimulatory effect of IGF-I on growth, as reviewed by Meinhardt and Ho (34). The observed dose-dependent reduction in body weight in the pubertal DES exposed animals in this study may thus be explained by estrogenic effects of DES on longitudinal growth and bone maturation. This effect was not seen when DES administration was initiated in adulthood. The effect seen on body weight in the pubertal animals is therefore considered to be an endocrine disrupting effect on growth and does not reflect systemic toxicity. Additionally, bone-length could be an interesting avenue to explore as endpoint for endocrine disruption by estrogenic mode of action.

In contrast to body weight, other endpoints showed higher sensitivity for adult than pubertal exposure. Decreased ovary weight was observed at a lower dose of DES with adult than pubertal exposure, and an increased adrenal weight was observed at a lower dose of KTZ with adult than pubertal exposure. The adult exposure scenario also appeared to be more sensitive in detecting effects of KTZ on ovaries, as adult exposure led to an increased ovary weight. Both the increase in ovary and adrenal weight caused by KTZ is related to its effects on cytochrome P450 (CYP) enzymes of the steroidogenesis pathway, particularly aromatase (CYP19A1) which is responsible for conversion of androgens to estrogens (13–15). Aromatase inhibitors are used for ovarian induction in fertility treatment of anovulatory women (35). A similar effect, stimulation of follicle growth by reduced levels of estradiol, and thereby reduced negative feedback on the pituitary, may have caused the increase in ovary weight and growing antral follicle numbers observed in the adult KTZ animals.

In the adult exposure scenario, KTZ-48 exposure increased the absolute liver weight by 26%. The increase could indicate adaptive changes after induction of cytochrome P450 (CYP) enzymes in the liver (36, 37), which is to be expected as ketoconazole has direct and specific effects on CYPs (13–15), including CYPs in the liver (38). The increased liver weight in the KTZ-48 exposure group is therefore likely not an indication of systemic toxicity, especially when considering that no reduction in body weight was observed, but rather related to the known mode of action of KTZ. The increase in liver weights compared to control in the KTZ-48 group (8% absolute increase) after pubertal exposure, as well as the increases seen in DES-0.012 (5% absolute increase) and DES-0.048 (3% absolute increase) groups after adult exposure are statistically significant, but small and a possible biological significance is questionable (36, 37).

Estrous cycling results were similar between exposure scenarios. Animals exposed to DES-0.012 and DES-0.048 showed clear effects with primarily prolonged cycles, seemingly due to persistent estrous in both exposure regimes. Persistent estrous after estrogen exposure early in life has previously been reported and is often related to polyfollicular anovulatory ovaries in adulthood, resembling polycystic ovarian syndrome (39). Interestingly, histological examination showed that animals exposed to DES during puberty had a reduced number of growing antral follicles as well as reduced corpus luteum number, indicating reduced ovulation. The same pattern was seen in animals exposed to DES-0.048 during adulthood. Hypothalamic GnRH secretion was not affected in these females (16). Unfortunately, we do not have measurements of luteinizing hormone/follicle stimulating hormone and it is therefore difficult to deduce if observed ovarian changes are associated with local and/or central effects of DES. The timing of exposure to KTZ did not seem to have an influence on estrous cycling as no statistically significant effects were registered for neither pubertal nor adult exposure.

## 5 Conclusion

The purpose of this study was to investigate if time of exposure affects the sensitivity of identification of (anti)estrogenic/(anti)androgenic effects in the 28-day study. We compared pubertal and adult exposure and found some differences in sensitivity for certain effect endpoints, but the differences were not very pronounced. A preference for pubertal exposure over adult exposure can therefore not be substantiated. However, the data indicates that timing of VO may be an endpoint particularly sensitive to estrogens and could function as a marker of endocrine disruption. We propose to continue the work on VO as well as the quest for novel biomarkers in blood, brain or locally in reproductive organs, as sensitive effect markers are warranted to detect female reproductive toxicants.

## Data availability statement

The original contributions presented in the study are included in the article/**Supplementary Material**. Further inquiries can be directed to the corresponding author.

## Ethics statement

The animal study was reviewed and approved by The Danish Animal Experiments Inspectorate (Council for Animal Experimentation, license number 2020-15-0201-00570) and monitored by DTU in-house Animal Welfare Committee.

## Author contributions

Study conception and design: JB, SC, TS, HJ; data collection: JB, HJ, MD; analysis and interpretation of results: JB, HJ, SC, MD, PD, TL. All authors reviewed the results and approved the final version of the manuscript.

## References

[1] van Duursen MBM Boberg J Christiansen S Connolly L Damdimopoulou P Filis P. Safeguarding female reproductive health against endocrine disrupting chemicals—The FREIA project. Int J Mol Sci (2020) 21(9):3215. doi: 10.3390/ijms21093215 32370092 PMC7246859

[2] Johansson HKL Christiansen S Draskau MK Svingen T Boberg J. Classical toxicity endpoints in female rats are insensitive to the human endocrine disruptors diethylstilbestrol and ketoconazole. Reprod Toxicol (2021) 101:9–17. doi: 10.1016/j.reprotox.2021.01.003 33571642

[3] OECD. Test no. 407: repeated dose 28-day oral toxicity study in rodents, OECD guidelines for the testing of chemicals, section 4. Paris: OECD Publishing (2008). doi: 10.1787/9789264070684-en

[4] OECD. Test no. 422: combined repeated dose toxicity study with the reproduction/developmental toxicity screening test, OECD guidelines for the testing of chemicals, section 4. Paris: OECD Publishing (2016). Available at: http://www.oecd.org/termsandconditions/. doi: 10.1787/9789264264403-en

[5] OECD. Report of the validation of the updated Test Guideline 407: Repeat dose 28-day oral toxicity study in laboratory rats, OECD Series on Testing and Assessment, No. 59. Paris: OECD (2006). Available at: https://www.oecd.org/officialdocuments/publicdisplaydocumentpdf/?cote=env/jm/mono(2006)26&doclanguage=en.

[6] OECD. Revised guidance document 150 on standardised test guidelines for evaluating chemicals for endocrine disruption, OECD series on testing and assessment, no. 150. Paris: OECD Publishing (2018). Available at: https://www.oecd-ilibrary.org/environment/guidance-document-on-standardised-test-guidelines-for-evaluating-chemicals-for-endocrine-disruption-2nd-edition_9789264304741-en. doi: 10.1787/9789264304741-en

[7] OECD. Guidance document on mammalian reproductive toxicity testing and assessment OECD. Environment, Health and Safety Publications. Series on testing and assessment. No. 43. Paris: OECD Publishing (2008).

[8] OECD. Guidance document for histologic evaluation of endocrine and reproductive tests in rodents. OECD series on Testing and Assessment, No 106. Paris: OECD Publishing (2009).

[9] Hoover RN Hyer M Pfeiffer RM Adam E Bond B Cheville AL. Adverse health outcomes in women exposed *in utero* to diethylstilbestrol. N Engl J Med (2011) 365(14):1304–14. doi: 10.1056/NEJMoa1013961 21991952

[10] Zamora-León P. Are the effects of DES over? A tragic lesson from the past. Int J Environ Res Public Health (2021) 18(19):10309. doi: 10.3390/ijerph181910309 34639609 PMC8507770

[11] Gal M Eldar-Geva T Margalioth EJ Barr I Orly J Diamant YZ. Attenuation of ovarian response by low-dose ketoconazole during superovulation in patients with polycystic ovary syndrome. Fertil Steril (1999) 72(1):26–31. doi: 10.1016/S0015-0282(99)00188-0 10428144

[12] Parsanezhad ME Alborzi S Pakniat M Schmidt EH. A double-blind, randomized, placebo-controlled study to assess the efficacy of ketoconazole for reducing the risk of ovarian hyperstimulation syndrome. Fertil Steril (2003) 80(5):1151–5. doi: 10.1016/S0015-0282(03)01177-4 14607566

[13] Kjærstad MB Taxvig C Nellemann C Vinggaard AM Andersen HR. Endocrine disrupting effects in *vitro* of conazole antifungals used as pesticides and pharmaceuticals. Reprod Toxicol (2010) 30(4):573–82. doi: 10.1016/j.reprotox.2010.07.009 20708073

[14] Munkboel CH Rasmussen TB Elgaard C Olesen MLK Kretschmann AC Styrishave B. The classic azole antifungal drugs are highly potent endocrine disruptors in vitro inhibiting steroidogenic CYP enzymes at concentrations lower than therapeutic Cmax. Toxicology (2019) 425(July):152247. doi: 10.1016/j.tox.2019.152247 31330226

[15] Mason JI Carr BR Murry BA. Imidazole antimycotics: Selective inhibitors of steroid aromatization and progesterone hydroxylation. Steroids (1987) 50:179–89. doi: 10.1016/0039-128X(83)90070-3 3504059

[16] Franssen D Johansson HKL Lopez-Rodriguez D Lavergne A Terwagne Q Boberg J. Perinatal exposure to the fungicide ketoconazole alters hypothalamic control of puberty in female rats. Front Endocrinol (Lausanne) (2023) 14:1140886/full(1140886). doi: 10.3389/fendo.2023.1140886/full 37077353 PMC10108553

[17] Kugathas I Johansson HKL Chan Sock Peng E Toupin M Evrard B Darde TA. Transcriptional profiling of the developing rat ovary following intrauterine exposure to the endocrine disruptors diethylstilbestrol and ketoconazole. Arch Toxicol (2023) 97(3):849–63. doi: 10.1007/s00204-023-03442-2 PMC9968686 36653537

[18] Li T Boberg J Johansson HKL Di Nisio V Christiansen S Svingen T. Quantitative analysis of ovarian surface photographs as a tool for assessment of chemical effects on folliculogenesis and ovulation in rats. Reprod Toxicol (2023) 119:108416. doi: 10.1016/j.reprotox.2023.108416 37268149

[19] Goldman JM Murr AS Cooper RL. The rodent estrous cycle: Characterization of vaginal cytology and its utility in toxicological studies. Birth Defects Res Part B - Dev Reprod Toxicol (2007) 80:84–97. doi: 10.1002/bdrb.20106 17342777

[20] Gaytan F Morales C Leon S Heras V Barroso A Avendaño MS. Development and validation of a method for precise dating of female puberty in laboratory rodents: The puberty ovarian maturation score (Pub-Score). Sci Rep (2017) 7(46381):srep46381. doi: 10.1038/srep46381 PMC5388887 28401948

[21] Hsu JC. The factor analytic approach to simultaneous inference in the general linear model. J Comput Graphical Stat (1992) 1(2):151–68. doi: 10.1081/E-EEE2-120046011

[22] Yang R Wang YM Zhang L Zhao ZM Zhao J Peng SQ. Prepubertal exposure to an oestrogenic mycotoxin zearalenone induces central precocious puberty in immature female rats through the mechanism of premature activation of hypothalamic kisspeptin-GPR54 signaling. Mol Cell Endocrinol (2016) 437:62–74. doi: 10.1016/j.mce.2016.08.012 27519634

[23] Dong W He J Wang J Sun W Sun Y Yu J. Bisphenol A exposure advances puberty onset by changing Kiss1 expression firstly in arcuate nucleus at juvenile period in female rats. Reprod Toxicol (2022) 110:141–9. doi: 10.1016/j.reprotox.2022.04.005 35429613

[24] Qiu J Sun Y Sun W Wang Y Fan T Yu J. Neonatal exposure to bisphenol A advances pubertal development in female rats. Mol Reprod Dev (2020) 87(4):503–11. doi: 10.1002/mrd.23329 32109339

[25] Thigpen JE Haseman JK Saunders HE Setchell KDR Grant MG Forsythe DB. Dietary phytoestrogens accelerate the time of vaginal opening in immature CD-1 mice. Comp Med (2003) 53(6):607–15. 14727808

[26] Nass TE Matt DW Judd HL Lu JKH. Prepubertal treatment with estrogen or testosterone precipitates the loss of regular estrous cyclicity and normal gonadotropin secretion in adult female rats. Biol Reprod (1984) 31(4):723–31. doi: 10.1095/biolreprod31.4.723 6439256

[27] Odum J Lefevre PA Tinwell H Van Miller JP Joiner RL Chapin RE. Comparison of the developmental and reproductive toxicity of diethylstilbestrol administered to rats in utero, lactationally, preweaning, or postweaning. Toxicol Sci (2002) 68(1):147–63. doi: 10.1093/toxsci/68.1.147 12075118

[28] Rogers RE Chai S Pask AJ Mattiske DM. Prenatal exposure to diethylstilbestrol has long-lasting, transgenerational impacts on fertility and reproductive development. Toxicol Sci (2023) 20:1–8. doi: 10.1093/toxsci/kfad066/7227122 PMC10464516 37471692

[29] Iwasa T Matsuzaki T Tungalagsuvd A Munkhzaya M Kuwahara A Yasui T. The advancement of the onset of vaginal opening in female rats subjected to chronic testosterone treatment occurs independently of hypothalamic Kiss1 and RFRP expression. Neuroendocrinol Lett (2015) 36(8):767–70. 26921577

[30] Lephart ED Mathews D Noble JF Ojeda SR. The Vaginal Epithelium of Immature Rats Metabolizes Androgens through and Aromatase-Like Reaction: Changes during the Time of Puberty. Biol Reprod (1989) 40:259–67. doi: 10.1095/biolreprod40.2.259 2720026

[31] Iwasa T Matsuzaki T Tungalagsuvd A Munkhzaya M Yiliyasi M Kato T. Effects of chronic DHEA treatment on central and peripheral reproductive parameters, the onset of vaginal opening and the estrous cycle in female rats. Gynecological Endocrinol (2016) 32(9):752–5. doi: 10.3109/09513590.2016.1163672 27019210

[32] Posobiec LM Vidal JD Hughes-Earle A Laffan SB Hart T. Early vaginal opening in juvenile female rats given BRAF-inhibitor dabrafenib is not associated with early physiologic sexual maturation. Birth Defects Res B Dev Reprod Toxicol (2015) 104(6):244–52. doi: 10.1002/bdrb.21165 26626128

[33] Nilsson O Marino R De Luca F Phillip M Baron J. Endocrine regulation of the growth plate. Horm Res Paediatr (2005) 64(4):157–65. doi: 10.1159/000088791 16205094

[34] Meinhardt UJ Ho KKY. Modulation of growth hormone action by sex steroids. Clin Endocrinol (Oxf) (2006) 65(4):413–22. doi: 10.1111/j.1365-2265.2006.02676.x 16984231

[35] Yang AM Cui N Sun YF Hao GM. Letrozole for female infertility. Front Endocrinol (Lausanne) (2021) 12:676133/full(676133). doi: 10.3389/fendo.2021.676133/full 34220713 PMC8245002

[36] Thoolen B Maronpot RR Harada T Nyska A Rousseaux C Nolte T. Proliferative and nonproliferative lesions of the rat and mouse hepatobiliary system. Toxicol Pathol (2010) 38(7_suppl):5S–81S. doi: 10.1177/0192623310386499 21191096

[37] Amacher DE Schomaker SJ Burkhardt JE. The relationship among microsomal enzyme induction, liver weight and histological change in rat toxicology studies. Food Chem Toxicol (1998) 36(9–10):831–9. doi: 10.1016/S0278-6915(98)00066-0 9737431

[38] Zhang W Ramamoorthy Y Kilicarslan T Nolte H Tyndale RF Sellers EM. Inhibition of cytochromes P450 by antifungal imidazole derivatives. Drug Metab Disposition (2002) 30(3):314–8. doi: 10.1124/dmd.30.3.314 11854151

[39] Singh KB. Persistent estrus rat models of polycystic ovary disease: An update. Fertil Steril (2005) 84(SUPPL. 2):1228–34. doi: 10.1016/j.fertnstert.2005.06.013 16210015

