# Supplementary material for: Comparison of female rat reproductive effects of pubertal versus adult exposure to known endocrine disruptors
Source: Front Endocrinol (Lausanne). 2023 Oct 3;14:1126485. doi: 10.3389/fendo.2023.1126485 (PMC10579898; doi:10.3389/fendo.2023.1126485)
Supplement: Supplementary file 1 [file DataSheet_1.pdf]

Supplementary Material

Comparison of female rat reproductive effects of pubertal *versus* adult exposure to known endocrine disruptors

Julie Boberg, Tianyi Li, Sofie Christiansen, Monica K. Draskau, Pauliina Damdimopoulou, Terje Svingen and Hanna K.L. Johansson

Supplementary Table 1. Follicle categories used in quantification.

Category	Area min (mm^2)	Area max (mm^2)
Small Follicle (SF)	-	0.06
Growing Antral Follicle (GA)	0.06	0.26
Pre-ovulatory Follicle (PO)	0.26	1.76
Corpus Luteum (CL)	-	-

1	M	D	P	Ø	ØM	MD	D	P	Ø	M	D		
1	M	M	D	P	Ø	Ø	M	MD	D	P	Ø	M	D
1	MD	DP	P	Ø	MT	D	P	Ø	MD	D			
1	M	D	P	Ø	M	T	D	P	Ø	M	M	D	-
1	DS	PØ	ØM	MD	D	P	Ø	M	D	D	P	ØM	MDT
1	DP	PØ	Ø	M	D	P	Ø	M	D	P	Ø	M	D
1	PØ	Ø	MD	D	PT	PØS	Ø	M	MD	DP	Ø	ØM	DP
1	DPØT	ØTT	ØM	MDT	D	P	ØT	ØM	MD	D	P	Ø	MDT
1	Ø	M	MD	DP	PØ	Ø	MD	MD	P	Ø	Ø	M	D
1	ØT	ØT	Ø	D	D	PS	Ø	M	MD	D	P	Ø	MT
1	Ø	MT	D	PS	PTT	PØ	Ø	ØM	ØMT	D	P	-	-
1	S	S	S	S	T	T	T	S	PØ	MD	-	-	-
2	M	DS	P	Ø	MD	D	DP	PØT	Ø	M	D	DPT	
2	M	D	P	Ø	ØM	MT	D	DPT	PØ	Ø	M	MD	D
2	D	P	Ø	M	MD	DS	P	Ø	M	D			
2	MD	DP	Ø	ØM	M	D	D	P	Ø	Ø	MD	MD	
2	MD	MD	MD	S	D	ØTT	MT	D	D	D	-	-	-
2	P	Ø	MD	MD	S	S	S	PS	ØS	MS	DS	PS	
2	PT	PØ	Ø	Ø	MD	D	D	P	Ø	Ø	M	D	
2	PT	PØ	ØMT	MDT	D	D	PØ	ØM	D	D	-	-	-
2	PT	Ø	M	MD	DPT	DPT	P	Ø	MT	D	DPT	-	-
2	ØM	M	MD	D	DS	P	ØT	MT	D	DPT	Ø	M	MDT
2	ØT	MT	MD	D	P	Ø	M	D	P	Ø	M	D	-
2	Ø	ØM	MT	D	D	DS	S	S	S	S	S	-	-
3	M	D	P	MT	PT	Ø	Ø	M	D	P	PØT		
3	MTT	DPT	PØT	M	D	PØ	ØM	MD	MDT	DPT			
3	P	Ø	MDT	Ø	ØT	ØMT	MDT	DPT	PT	PØ	ØT	MT	
3	P	P	P	ØMT	MD	P	ØMT	ØMT	M	P	Ø	M	MD
3	PØ	Ø	Ø	ØT	ØT	ØM	MT	PØT	MD	D	-	-	-
3	ØTT	MD	MD	S	PS	ØT	MT	D	PS	P	PØ	ØM	D
3	ØT	ØTT	ØTT	ØTT	PØ	ØM	M	MD	P	Ø	M	D	
3	ØT	ØM	ØM	ØMT	MT	DP	DPT	DPT	ØT	MT	-	-	-
3	ØT	Ø	ØT	ØT	ØT	ØMT	PTT	ØMT	MDT	PTT	Ø	MDT	PT
3	Ø	PT	Ø	Ø	Ø	Ø	MT	M	D	S	S	P	MD
3	Ø	Ø	Ø	Ø	MPØ	Ø	Ø	MØP?	Ø	Ø	ØMT	P	-
3	Ø	Ø	Ø	ØMT	DPT	P	PØT	MD	DPT	DPT	-	-	-
4	P	Ø	Ø	Ø	ØMD	PØ	MD	Ø	Ø	Ø	Ø	M	
4	PØT	PØT	PØT	ØMDT	PØT	ØT	ØMT	MT	PØT	MDT	-	-	-
4	Ø	Ø	ØM	Ø	Ø	PØ	Ø	Ø	Ø	Ø	Ø		
4	Ø	Ø	Ø	Ø	PØ	Ø	Ø	Ø	Ø	Ø	Ø	Ø	Ø
4	Ø	Ø	Ø	Ø	PØ	Ø	Ø	Ø	PØ	M			
4	Ø	Ø	Ø	Ø	ØM	P	Ø	Ø	Ø	Ø	Ø		
4	Ø	PØ	P	Ø	Ø	Ø	Ø	Ø	Ø	Ø	Ø	Ø	
4	ØT	Ø	Ø	Ø	M	DPT	PØT	MDT	T	PT	-	-	-
4	ØTT	ØMT	ØT	Ø	Ø	Ø	Ø	ØM	PØTT	Ø	Ø	Ø	ØT
4	MØT	PØT	PØT	Ø	MT	DPT	PT	PØT	Ø	Ø	ØMT	P	-
4	Ø	Ø	Ø	Ø	Ø	Ø	ØM	DP	ØT	Ø	M	PØ	-
4	Ø	Ø	Ø	Ø	Ø	Ø	Ø	PØ	ØM	Ø	MP	MD	-

Supplementary Figure 1. Pubertal exposure. Estrous cycle data for individual rats exposed to DES or vehicle for 28 days starting PND 23. Group 1 corresponds to control, group 2 to DES-0.003, group 3 to DES-0.012 and group 4 to DES-0.048.

	70	71	72	73	74	75	76	77	78	79	80	81	82	83	84	85	86	87	88	89	90	91	92	93	94
1	D	DS	P	Ø	M	D	S	S	S	D	S	SØ	SM	SD	SP	ØT	M	P	PØT	PØT	Ø	Ø	M	D	
1	M	D	P	Ø	ØM	M	M	D	PT	P	Ø	ØM	MT	D	P	Ø	M	D	P	P	Ø	M	MD		
1	D	P	Ø	ØM	M	D	P	Ø	ØM	MT	D	P	Ø	ØM	M	PØ	ØM	D	P	T	ØM	P			
1	D	PØ	ØM	MT	DP	PØ	Ø	MT	MD	P	Ø	M	D	P	Ø	MD	M	D	P	Ø	M	D			
1	SP	PØ	M	D	PT	ØS	MT	MDS	S	S	T	ST	S	P	ØM	MDT	DPT	PØ	ØM	PS	MS	MS	PS	PS	ØM
1	P	Ø	M	MD	D	P	Ø	MT	D	P	Ø	MD	D	P	Ø	M	D	P	MD	M	D	P	Ø	M	D
1	Ø	M	D	P	Ø	MT	D	PØ	Ø	ØM	M	D	P	PØ	ØM	D	P	Ø	M	D	P	Ø	MD	DP	
1	ØS	MT	MD	D	P	PØT	MT	D	PØ	ØM	MT	D	PT	PØ	MD	D	PØ	PØ	M	D	Ø	Ø	MDT	MDT	
1	Ø	M	D	D	S	S	DS	S	ØS	S	S	S	S	S	S	ØST	M	MD	D	P	Ø	MT	D		
1	ØM	MT	D	SP	Ø	Ø	MD	MD	D	P	Ø	MT	D	P	Ø	M	MDT	D	PS	Ø	M	MD			
1	Ø	ØM	MT	MT	PT	Ø	M	MD	D	P	Ø	MT	D	P	Ø	D	D	P	Ø	M	D	PØT			
1	S	S	S	MS	S	PS	ØS	MDT	PØ	M	D	PS	S	S	S	PT	S	S	S	PS	ØM	PT			
2	MD	D	PTT	Ø	ØM	DPT	PT	ØTT	Ø	ØTT	M	P	P	M	DPT	Ø	Ø	MT	D	PTT	Ø	Ø	M		
2	M	D	P	Ø	ØM	MD	D	PS	Ø	Ø	M	D	P	ØT	ØM	M	D	PT	Ø	Ø	M	D			
2	D	P	Ø	MT	D	ST	P	Ø	MT	D	DP	P	ØT	MT	D	PS	PØ	Ø	M	MD	DS	S	ST	Ø	MT
2	DS	P	Ø	ST	D	S	P	ØST	MT	D	PST	ØS	ØMS	D	DPT	DPT	P	ØMST	MT	D	ØTT	Ø	ØTT		
2	PTT	Ø	Ø	M	MD	D	Ø	Ø	ØM	M	D	D	S	P	Ø	M	D	PT	Ø	Ø	ØM	M	D		
2	PØ	ØMD	MT	D	PS	Ø	ØM	MDT	MDT	DS	Ø	M	MD	DS	D	P	PØ	M	MD	D	PØ	ØM	D		
2	Ø	M	D	DPT	PT	ØS	MT	D	PØ	ØM	D	DPT	PØT	Ø	M	MT	D	PT	Ø	M	MD	D	P	Ø	MT
2	Ø	ØMD	MST	DT	PT	Ø	ØM	MTT	D	ST	S	S	Ø	M	D	P	Ø	M	D	P	Ø	MD	DT		
2	S	S	S	S	S	S	S	S	S	MT	S	S	S	S	S	DT	PØ	MD	DS	S	P	Ø	MD	D	
2	S	P	Ø	MD	PØ	MT	PØ	Ø	Ø	ØM	MT	MT	DPT	PT	S	Ø	ØM	M	D	DS	Ø	Ø	MT	PØ	M
2	S	P	Ø	M	D	P	Ø	M	MT	D	D	Ø	ØM	M	D	PT	Ø	ØM	MD	DP	P	P	Ø	MD	DPT
2	T	Ø	M	D	D	S	PØ	ØM	MT	DS	P	Ø	MT	MT	D	P	Ø	M	M	D	PTT	Ø	ØM	MDT	DT
3	MT	D	ST	ST	PØ	MT	MT	D	P	Ø	M	D	T	PØ	M	M	D	DP	Ø	ØMD	MDT	DT	PTT	Ø	MDT
3	ØMT	MTT	MT	DPT	PT	ØT	M	MD	DPT	PT	Ø	M	DPT	DPT	PØ	ØT	M	M	D	P	Ø	ØM	MT		
3	Ø	MST	D	ST	S	ST	PØ	ØMS	ØMT	DS	PØ	PØ	ØM	MT	ØMT	MDS	DPS	ØT	Ø	Ø	ØTT	MTT	MST	MST	DST
3	ØMT	MT	DT	ST	PS	PØST	ØMST	MTT	S	S	PØ	M	MS	MDS	DS	PØS	ØMST	MT	M	MST	S	S	ST		
3	Ø	M	D	T	PST	PS	PØS	ØM	MT	MDT	DP	PØ	Ø	M	MD	P	P	Ø	ØT	ØM	MD	T	T		
3	S	PØ	MD	T	ST	S	S	ØM	MT	MDT	DPT	ØTT	ØMTT	MTT	MDT	D	PST	ØS	MT	MDS	PØTT	PØSTT	PØST	MT	MDT
3	S	P	PØT	MT	MDT	PT	S	PØ	Ø	ØM	D	MD	S	S	SØ	M	P	M	MDT	ØMDT	PST	S	S		
3	ØT	Ø	MT	ØM	PT	PS	Ø	Ø	M	ØM	M	DP	S	S	S	S	S	S	S	S	S	S	S		
3	ØMTT	ØMT	MD	PST	ØT	MT	MT	D	PØ	ØMT	MT	MD	DPT	PØ	ØMT	ØMT	ØMT	D	P	Ø	MT	ØM	D		
3	S	ØM	M	MD	D	DP	PØ	ØMT	MS	ST	PØ	Ø	M	MD	PØ	Ø	Ø	Ø	MT	ØMD	PTT	P	PØ		
3	ST	S	ØT	MTT	MTT	PØT	MT	ST	PØS	ØMT	M	DT	T	PST	ØMT	MT	MDT	D	PØ	ØMT	MTT	D			
3	S	S	ST	S	ST	ST	S	S	S	DS	D	PØ	M	D	S	ØSTT	P	ØM	M	D	P	S	MS	M	
4	MDS	MDS	DPT	PTT	PST	PST	P	ØTT	ØMT	ØMT	MT	PØT	PØT	ØMT	ØMT	PØT	PØT	ØT	ØT	ØMT	ØMT	ØMT			
4	MD	D	S	S	S	ST	S	S	S	S	ST	S	S	ØMS	MS	MS	S	MST	MST	PST	PST	DPS	PS	DPS	
4	MD	DPST	S	S	S	S	S	S	S	ST	ST	MS	ØMS	ØMS	ØMS	ØMS	PØS	Ø	ØM	ØT	ØST	MT	Ø	ØM	
4	MT	PST	S	S	S	ST	ST	S	S	S	S	S	T	S	PSTT	PSTT	PØMST	S	PMST	PØMS	S	PST	ØTT	MTT	
4	MD	S	S	S	S	S	S	S	S	S	S	S	S	S	PS	ØMS	ØMT	ØMT	DPT	ØT	Ø	Ø	Ø	ØT	Ø
4	PT	PS	S	ST	S	S	S	S	S	S	T	S	S	S	S	S	S	S	S	S	S	ST	SPT	PS	Ø
4	PT	S	S	S	S	S	S	S	ØMST	ØMST	PS	STT	ØM	M	MDT	P	Ø	ØM	Ø	ØM	ØM	ØM	Ø	ØMT	
4	PT	S	S	S	S	ST	ØSTT	MS	PTTS	PSTT	ØMST	S	S	ST	MSTT	ØSTT	ØMSTT	PSTT	Ø	MST	T	T	ST		
4	S	PST	ØST	MST	PST	S	ØST	ØST	S	ST	PS	Ø	Ø	Ø	Ø	Ø	Ø	Ø	Ø	Ø	Ø	Ø	ØMT	MT	
4	S	S	S	S	S	ST	ST	S	S	ST	DT	S	S	S	S	S	S	S	ST	PØT	MST	MST			
4	S	S	S	S	S	S	ØMST	S	S	ST	ØMST	ØMS	ØMST	S	S	STT	STT	ØMT	MT	PØS	Ø	ØST			
4	ST	S	S	S	S	PS	PØST	ØMST	ØMST	PØST	Ø	ØT	ØTT	Ø	Ø	M	ØT	ØT	Ø	Ø	ØTT	ØT	Ø	ØTT	

Supplementary Figure 2. Adult exposure. Estrous cycle data for individual rats exposed to DES or vehicle for 28 days starting PND 63. Group 1 corresponds to control, group 2 to DES-0.003, group 3 to DES-0.012 and group 4 to DES-0.048.
